# Portable colorimetric enzymatic disposable biosensor for histamine and simultaneous histamine/tyramine determination using a smartphone

**DOI:** 10.1007/s00216-023-04583-0

**Published:** 2023-02-15

**Authors:** Isabel Sanz-Vicente, Irina Rivero, Lucía Marcuello, María Pilar Montano, Susana de Marcos, Javier Galbán

**Affiliations:** 1 Nanosensors and Bioanalytical Systems (N&SB), Analytical Chemistry Department, Faculty of Sciences, Aragon Institute of Nanoscience, University of Zaragoza, 50009 Saragossa, Spain; 2 Analytical Chemistry Department, Faculty of Sciences, University of Zaragoza, 50009 Saragossa, Spain

**Keywords:** Histamine, Tyramine, Tyramine oxidase, Amplex red, Disposable biosensor, Cellulose

## Abstract

Tyramine oxidase (TAO), peroxidase (HRP), and Amplex Red (AR) have been immobilized on cellulose to obtain disposable biosensors for the determination of histamine. During the enzymatic reaction, AR is oxidized and a pink spot is obtained. Using a smartphone and measuring the G (green) color coordinate, histamine can be determined in the presence of other biogenic amines (putrescine and cadaverine) in concentrations ranging from 2·10^−5^ M to 5·10^−4^ M with a 7.5·10^−6^ M limit of detection (LoD). Despite tyramine interference, experimental conditions are provided which allow rapid and simple histamine and simultaneous histamine/tyramine (semi)quantitative determination in mixtures. Finally, tyramine and histamine were determined in a tuna extract with good results (compared to the reference HPLC–MS method). The methodology can also be applied in solution allowing histamine (and simultaneous histamine/tyramine) determination with a lower LoD (1.8·10^−7^ M) and a similar selectivity.

**Graphical abstract:**

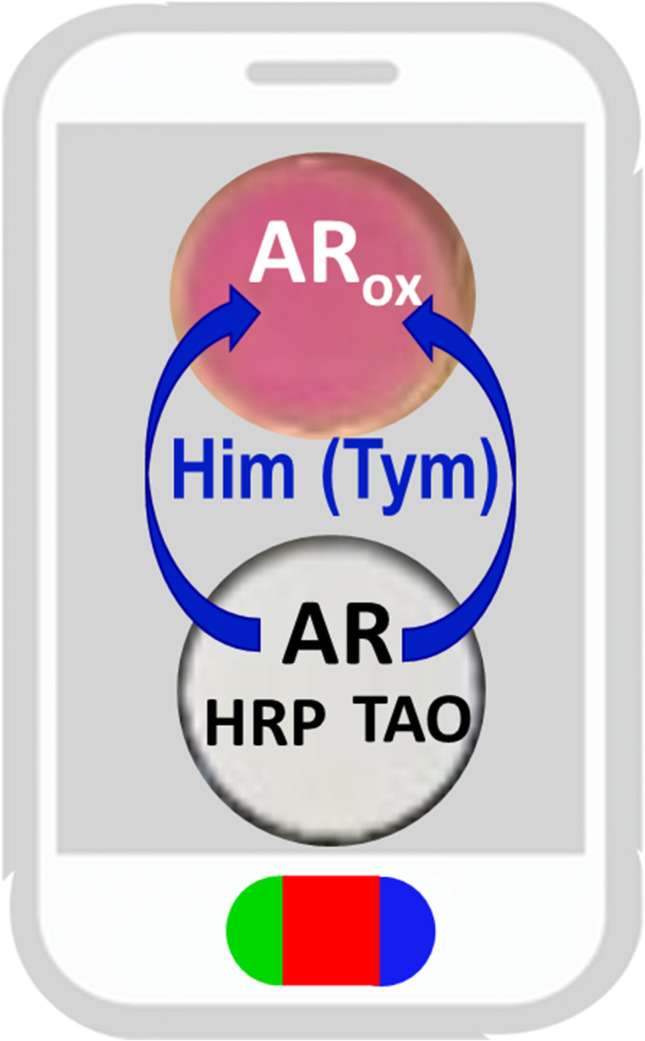

**Supplementary Information:**

The online version contains supplementary material available at 10.1007/s00216-023-04583-0.

## Introduction

Histamine is a low molecular weight biogenic amine (BA) which plays two opposite roles in the human body. At low concentrations, it is essential because it acts as a neurotransmitter [1], a regulator of the circulatory system [2], and it takes part in inflammatory processes [3]. At high concentrations, it becomes a toxic compound producing adverse symptoms in the organism [4].

BAs are mainly present in food. They are formed by the enzymatic decarboxylation of the corresponding amino acid (histidine in the case of histamine, or tyrosine in the case of tyramine) [5, 6]. Particularly, the highest histamine levels are found in fermented foods (wine, beer, or cheese) [7] and those having high concentrations of proteins such as meat and fish (especially scombrids like tuna). The ingestion of foods rich in histamine increases its concentration in the body, leading to intolerance or intoxication [8, 9] (that due to histamine is known as scombroid poisoning [10]). The relevance of this problem has prompted the European Union to establish histamine limits of 200 and 400 mg/kg in fresh and canned fish, respectively [11]. To eliminate the risk associated with histamine poisoning, storing raw materials or foods at low temperatures has been tested. This prevents bacterial growth; however, some types of bacteria can grow at low temperatures and form histidine decarboxylase [12]. Cooking can deactivate the action of enzymes and microorganisms, but it does not eliminate histamine already formed [13] because it is a thermally stable compound.

From the analytical point of view, the determination of BAs in foods is not easy due to the chemical complexity of the matrixes, the variable concentration ranges, the simultaneous presence of many BAs, additional interferences, and the absence of intrinsic analytical properties of these compounds [14]. Their quantification is mainly based on separation techniques, HPLC being the technique of choice, especially to determine histamine [14, 15]. Based on this technique, methods with good analytical figures of merit have been proposed for histamine; however, they are neither fast enough, nor simple and portable for in situ control, which is mandatory when commercial spoiled foods need to be detected.

Lately, spectrophotometric strategies are emerging [16] based on the effect of histamine on the optical properties of nanoparticles, such as color change of AuNPs by aggregation [17, 18], quenching in the fluorescence of quantum dots [19], or d-penicillamine capped copper nanoparticles [20]. However, selectivity to histamine in the presence of other BAs is better guaranteed using immunoassays or enzymatic methods.

Several commercial immunoassays for histamine are available (e.g., Veratox Histamine™, Histasure™, HistaQuant™, or HistaMeter™). These are capable of determining concentrations in the range from 2.5 to 250 mg/L, but analysis times are very long (30–90 min apart from the sample preparation time) [21] and the cost is not competitive for daily testing.

**Fig. 1:**
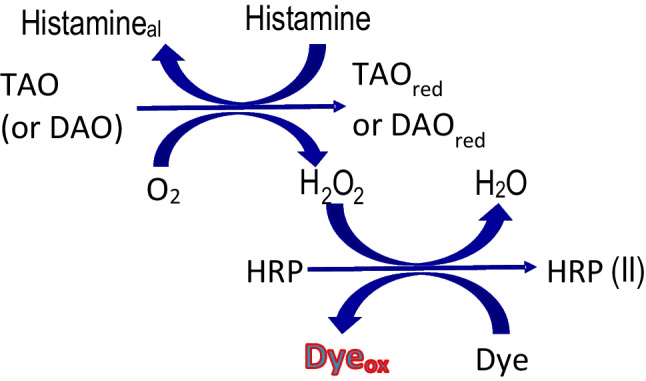
Sequence of enzymatic reactions

So far, enzymatic methods for histamine have mainly been based on one of the two following enzymes: (A) histamine dehydrogenase (HDHA) [22], which in the presence of 1-methoxy-5-methylphenazinium methylsulfate (1-methoxy PMS) gives a formazan dye that absorbs at 492 nm; (B) diamine oxidase (DAO) which catalyses the histamine oxidation to imidazole acetaldehyde (Histamine_al_), ammonia, and hydrogen peroxide (Fig. 1). The consumed O_2_ [23] or, better, the formed H_2_O_2_ can be measured; in the latter case, by coupling a second enzymatic reaction involving a chromogen and the enzyme peroxidase [24, 25]. The most important problems with these methods include the instability of the chromogen and the interferences caused by other BAs; for these reasons, alternatives are being proposed [26, 27] which involve more stable chromogens or more selective enzymes. To simplify the application of the method and make it more competitive, a very interesting option is the development of disposable biosensors based on test strips prepared by immobilization of enzymes on paper or cellulose [28]. Following this idea, Hall [29] dipped commercial peroxide test strips in a mixture of DAO and HRP, making them sensitive to histamine. As far as we know, only HDHA-based enzymatic tests for histamine are commercially available (Kikkoman Biochemifa Company™ and Megazyme histamine assay kit (K-HISTA)™). These require incubation times of 20 min at 37 °C and are very prone to the interference by reducing chemicals [30].

In our research group, enzymatic colorimetric methods are being developed to determine BAs. Using DAO, cadaverine and putrescine have been determined (using 2,2′-Azino-bis (3-ethylbenzothiazoline-6-sulfonic acid) diammonium salt (ABTS) or 3,3′,5,5′-tetramethylbenzidine (TMB) as dyes) [31, 32]. Using tyramine oxidase (TAO), tyramine [33] has been determined both in solution and on cellulose supports. In both cases, histamine was a two-way interference: first, it is a DAO and a TAO subtract, and second, more importantly, Histamine_al_ is able to reduce the colored oxidized dye (Dye_ox_, in Fig. 1).

The continuous technological improvements in smartphones (better cameras, light sources, and software) are allowing these devices to be increasingly used in analytical chemistry for qualitative and semiquantitative analyses [34, 35].

In this work, we propose an enzymatic colorimetric analytical system which overcomes these problems. First, TAO is proposed (for the first time) as the enzyme, instead of DAO or HDHA, and the appropriate experimental reaction conditions are carefully chosen. Second, Amplex Red® is used as the dye, which Dye_ox_ does not react with Histamine_al_. The method has been characterized first in solution, and later, it has been implemented on supports made of powdered cellulose for the fast on-site determination of histamine (less than 2 min) using a smartphone (taking advantage of the RGB readout facility of the camera). This has made the methodology fully portable and accessible to any user without specific training. On the other hand, and due to the small dimensions of the biosensors developed, the consumption of reagents is low, which is in line with the green chemistry principles, and the test is very cheap (one test less than 0.50 €). Finally, experimental conditions have also been studied for the simultaneous determination of histamine and tyramine.

## Material and methods

### Reagents and solutions

Phosphate buffer solutions (0.1 M, pH 6.0, 7.0, and 8.0) were prepared from Na_2_HPO_4_ and NaH_2_PO_4_ solids (Sigma S9638 and S9763). Carbonate buffer solution (0.1 M, pH 9.0) was prepared from Na_2_CO_3_ (Sigma 222,321) and NaHCO_3_ (Sigma S5761).

Hydrogen peroxide stock solution (33% w/v) was supplied by Panreac (131,077.1211); the exact concentration was established and periodically checked by titration using potassium permanganate (oxalic acid as primary standard). Peroxidase from *Horseradish* (HRP EC 1.11.1.7) was obtained from Sigma (P8125 88.6 U mg^−1^). Diamine oxidase from *Lathirus cicera* 280 U mL^−1^ (DAO EC 1.4.3.22) was purchased from Molirom P021. Tyramine oxidase (TAO EC 1.4.3.9) from *Arthrobacter* sp. (T-25) 4600 U mg^−1^ was purchased from Asahi Kasei Pharma Corporation.

Cadaverine (C8561), putrescine (P7505), histamine (53,300), tyramine (T287998), 2,2′-Azino-bis (3-ethylbenzothiazoline-6-sulfonic acid) diammonium salt (ABTS) (A1888), 10-Acetyl-3,7-dihydroxyphenoxazine (Amplex Red™, AR) (90,101), and 3,3′,5,5′-Tetramethylbenzidine (TMB) (860,336) were supplied by Sigma. All solutions were daily prepared by weighing and dissolving in the buffer solution (minus TMB and AR, which was dissolved in dimethyl sulfoxide (Panreac131954.1611)). TMB, ABTS, and AR solutions were stored in darkness.

Cellulose microcrystalline of 20 μm of particle size and average degree of polymerization minor than 350 (Aldrich 310,697) was used to develop the biosensors.

### Equipments and instruments

Molecular absorption measurements were performed using a Hewlett-Packard model HP 8452A diode-array spectrophotometer equipped with a HP 89090A Peltier temperature and stirrer control accessory. Depending on the measurement wavelength, quartz (Hellma QS 101) or glass (Hellma Q 101) cuvettes were used.

Cellulose supports were dried in an OVAN incubator model OM10E.

A smartphone Xiaomi Redmi Note 8 Plus (64 Mpixels) was used to measure the color development in the cellulose biosensors. The application used to capture the RGB coordinates was Color Grab™ (from Loomatrix).

### Procedure

#### Measurements in solution

The procedure is similar to that described in previous articles [31]. The variation of the absorbance during the enzymatic reaction was measured at different wavelengths depending on the dye used: 570 nm (AR), 650 nm (TMB) and 730 nm (ABTS). To do so, the appropriate concentration of the reagents (HRP, DAO or TAO and dye) was added to the cuvette with the buffer solution. The total volume in the cell was 2 mL. The cuvette was then placed in the spectrophotometer, the stirrer was connected, and the measurement was started in the kinetic mode. After a few seconds (to obtain the baseline), 20 μL of the analyte (or sample) solution was injected and the variation of the absorbance during the reaction was recorded over the time. As the diode-array spectrophotometer has a reverse optic configuration, a yellow filter must be placed between the lamp (D_2_) and the cell to avoid the eventual photooxidation of the dye. During the optimization studies, the concentration of the reagents and other conditions were modified in line with the parameter studied. The maximum absorbance at the chosen wavelength (Abs_max,λ_) was used as the analytical parameter.

#### Biosensor preparation

The template used was the lid of a conventional 96-well plate. A 5% (w/V) water dispersion of cellulose containing AR (2·10^−4^ M) was prepared. Seventy-five microliters of this mixture was added to the template wells (it has small circles that act as stops), placed in the incubator and dried at 35 °C for 1 h (cellulose support). Then, 10 μL of an enzyme mixture (23 U mL^−1^ TAO and 20 U mL^−1^ HRP) was added (biosensor) and, after 30 s, 10 μL of the analyte solution.

#### Measurements using the smartphone

To maintain constant lighting conditions, the measurements were taken in an area of the laboratory previously conditioned for color measurements, namely, with the same lamp (fluorescent) and the smartphone placed at the same height with regard to the samples (see scheme in ESM, Fig. S1). The analytical biosensors were moved under it. The reference RGB values were first taken before the addition of the analyte (named R_0_, G_0_, B_0_, respectively). Later, the analyte was added and the RGB values were obtained again (named R, G and B). The analytical signals were ΔR (R_0_ − R), ΔG (G_0_ − G) and ΔB (B_0_ − B). The tool of the software (Color Grab™ from Loomatrix) for taking the RGB values at a located area of the smartphone screen was chosen. This tool allows the user to take color measurements “in situ.” Due to the fact that the color signal that the sensor receives goes directly to the devices screen (displaying colors in sRGB color space), parameters related to image capture, such as ISO or exposure time, cannot be selected. However, the white balance option is enabled, allowing color correction for most standard illuminants.

#### Analytical characteristics

Throughout the article, precision values have been expressed as the standard deviation of the corresponding replicates (sd).

During the optimization studies, all measurements were performed a minimum of three times.

The limit of detection (LoD) has been calculated as three times the standard deviation of the blank signal divided by the sensitivity (the slope as the linear part of the calibration line).

The errors made when obtaining the concentrations are given as relative errors (%).

### Tuna sample treatment

A tuna extract (from a local supermarket) was prepared and analyzed by HPLC–MS by the Laboratorio de Salud Pública of Aragón (LSPA) using a previously validated method [36]. 2.5 g tuna were treated with 20 mL 5% trichloroacetic acid; the samples were shaken in a vortex for 30 s. Then, the mixture was submitted to ultracentrifugation for 10 min at 4000 rpm (4 °C); this operation was repeated twice. The filtrated was taken to 50 mL.

The following concentrations were obtained (found ± sd, in mg kg^−1^): 100 ± 11 putrescine, 380 ± 19 cadaverine, 900 ± 40 histamine, 300 ± 22 tyramine.

A fraction of this extract was analyzed by the procedure previously described.

## Results and discussion

### Method in solution

#### Chromogen selection

**Fig. 2:**
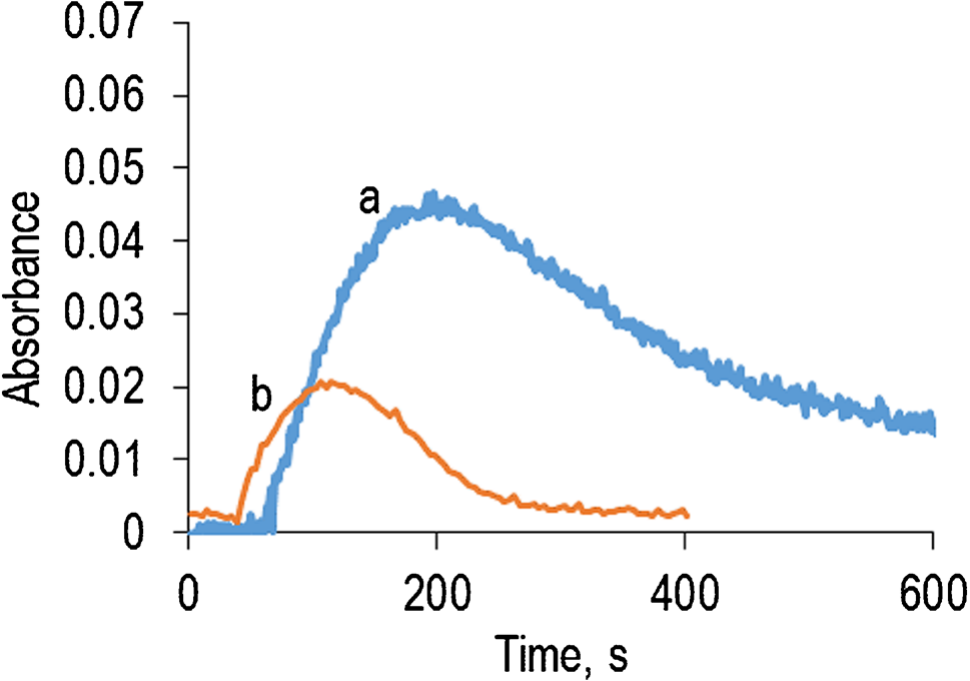
Absorbance variation of the dyes during the reaction. Experimental conditions: (a) [TMB] = 6·10^−5^ M, [HRP] = 0.5 U mL^−1^, [DAO] = 1.8 U mL ^−1^, [Histamine] = 4·10^−5^ M, pH = 6, λ = 650 nm. (b) [ABTS] = 4.4·10^−5^ M, [HRP] = 2 U mL^−1^, [DAO] = 1.8 U mL^−1^, [Histamine] = 2.4·10^−5^ M, pH = 6, *λ* = 730 nm

The analytical system developed in this study is based on the sequence of the enzymatic reactions shown in Fig. 1. In this type of method, TMB is a frequently used chromogen. As is very well known, the original chemical form of this dye (reduced, TMB) is colorless and the oxidized form (TMB_ox_) is blue, which allows us to obtain high sensitivity for H_2_O_2_ determination. An important drawback is that TMB_ox_ suffers several lateral reactions [31] which cause the obtained blue color to vanish, the most important being its reaction with reducing aminoacids (such as tyrosine, tryptophan, or cysteine which are placed in the outer sphere of proteins), according to the general scheme:

1 $${\mathrm{TMB}}_{\mathrm{OX}}+\mathrm{Protein}\rightleftharpoons \mathrm{TMB}+{\mathrm{Protein}}_{\left(\mathrm{OX}\right)}$$

(if proteins are enzymes, this reaction does not affect their catalytic properties). This problem can be partially solved by forcing the equilibrium (1) shifting to the left by working with higher TMB concentrations. However, when TMB was tested for histamine determination according to the scheme of Fig. 1, an additional problem appeared (Fig. 2, line a): the absorbance increased up to a maximum and later decreased down to the initial absorbance (zero). After several studies, we concluded that Histamine_al_ regenerates the TMB_ox_ (similar to that shown in (1)). This impedes the use of TMB for this determination. ABTS, another dye frequently used in this type of method, gave the same problem (Fig. 2, line b).

New chromogens were tested, and finally, good results were obtained with Amplex Red™ (AR). During the enzymatic reaction, AR is oxidized to resorufin (AR_ox_) which can be measured by spectrophotometry (*λ*_max_ = 570 nm, ε ~ 60,000 M^–1^ cm^–1^) with high sensitivity (see ESM, Fig. S2). An optimization study of the HRP/H_2_O_2_/AR indicating reaction was carried out and is shown in ESM. The maximum absorbance was obtained using a 4·10^−6^ M dye concentration (ESM, Fig. S3), from which it remains constant. Regarding the HRP concentration (ESM, Fig. S4), the maximum absorbance value was obtained using 0.2 U mL^−1^. In the best conditions, a calibration line was obtained (ESM, Fig. S5), being linear up to at least 2·10^−5^ M (maximum concentration tested).

#### Enzyme selection

DAO and TAO belong to the amine oxidase copper-containing enzymes family; both enzymes have the same active center but present different selectivity. DAO is more appropriate for diamines (such as putrescine or cadaverine) but it also reacts with histamine (in fact, it is sometimes called histaminase). TAO is more appropriate for tyramine but it also shows some activity towards other amines. Neither of the two is specific (or selective) to histamine, so it is interesting to compare their ability towards this compound.

**Fig. 3:**
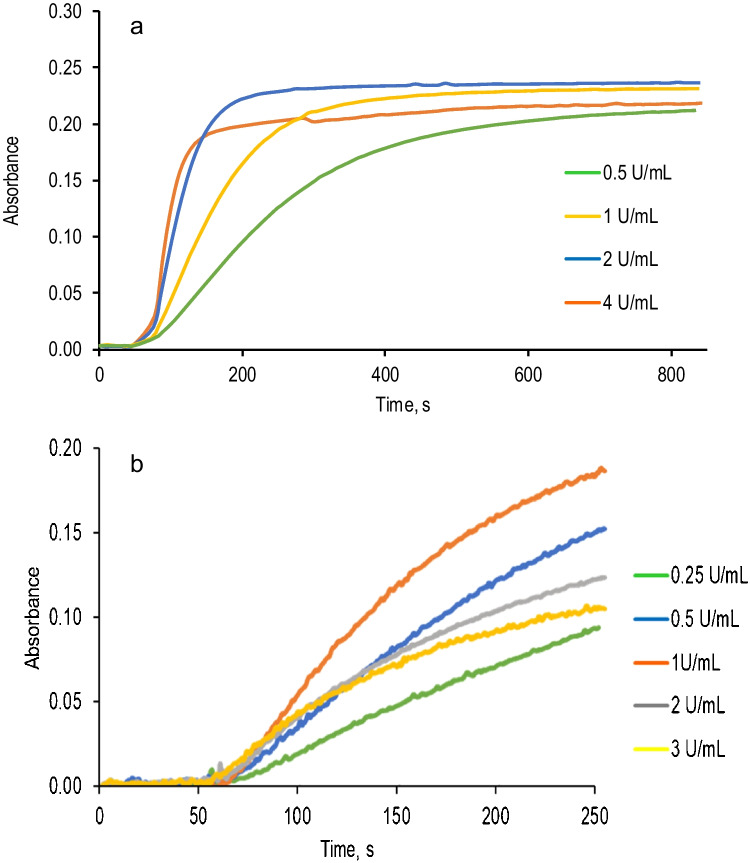
Optimization of the enzyme concentration**. a** DAO. Experimental conditions: [AR] = 7·10^−5^ M, [HRP] = 0.1 U mL^−1^, [Histamine] = 5·10^−6^ M, *λ* = 570 nm, pH = 8. **b** TAO. Experimental conditions: [AR] = 7·10^−5^ M, [HRP] = 0.1 U mL^−1^, [Histamine] = 5·10^−6^ M, *λ* = 570 nm, pH = 8

The most important parameters to be optimized for this determination are the pH and enzyme concentration. The pH is crucial, not only for the formation of AR_ox_ and to control the activity of both enzymes, but also to modulate the interference level of other BA (see below). Fig. S6 shows the effect of pH (in the range 6 to 10) in the absorbance at 570 nm for Histamine determination using DAO (a) and TAO (b). The best pH to determine histamine in both cases was 8–9; outside these values, the enzyme activity towards histamine (specially TAO) sharply decreases. Figure 3 shows the absorbance *vs* time profiles obtained for different concentrations of enzymes. As can be seen, both the kinetic of the reaction and the maximum absorbance increase with the concentration up to a maximum (2 U mL^−1^ for DAO (Fig. 3a) and 1 U mL^−1^ for TAO (Fig. 3b)). The decrease in sensitivity observed at high concentrations can be explained by the process (1), which indicates that the outer aminoacids of both enzymes are able to react with AR_ox_. Since the extent of this process depends on the aminoacids configuration of each particular enzyme, TAO is more prone than DAO to react with AR_ox_. The effect of these enzymes can be softened by increasing the AR concentration; in any event, AR proved to be less prone than ABTS or TMB to this reaction. Besides the pH and enzyme concentration, the AR and HRP concentrations were also optimized. The results are compiled in ESM. The HRP concentration effect was studied in the range from 0.01 to 2.0 U mL^−1^ (Table S1). Concentrations equal to or greater than 0.10 U mL^−1^ did not modify the kinetic of the whole reaction indicating that amine oxidase is the enzyme that controls the process. The AR concentration was studied in the range from 9·10^−6^ M to 9·10^−5^ M. As can be seen (Fig. S7), AR did not affect the signal obtained very much (7·10^−5^ M was final chosen as optimum concentration).

#### Analytical figures of merit. Interferences

In the optimum conditions found, Table 1 gives the linear response range (see ESM; Fig. S8a is the calibration line using DAO and Fig. S8b using TAO), the sensitivity, LoD, and RSD (%) for histamine determination using both enzymes. Considering that one histamine molecule should produce one H_2_O_2_ molecule, the sensitivity (slope of the calibration line) for histamine should be the same as the sensitivity for H_2_O_2_ (Fig. S5). By calculating the relative histamine/H_2_O_2_ sensitivity (slopes of the corresponding calibration lines), the % histamine conversion during the enzymatic reaction for both enzymes has been calculated; as can be seen, 74% and 63% were obtained for DAO and TAO, respectively.

**Table 1:** Analytical figures of merit for histamine. Experimental conditions: [HRP] = 0.1 U mL^−1^, [AR] = 7·10^−5^ M, *λ* = 570 nm, pH = 8

Enzyme	Range, M	Sensitivity, M^−1^	LoD, M	RSD, % (*n*)
[DAO] = 2 U mL^−1^	4.6·10^−7^–8.4·10^−6^	6.13·10^4^	1.4·10^−7^	3.2 (5)
[TAO] = 1 U mL^−1^	6.1·10^−7^–1.6·10^−5^	5.16·10^4^	1.8·10^−7^	1.3 (5)

*LoD* limit of detection, *RSD* relative standard deviation

**Fig. 4:**
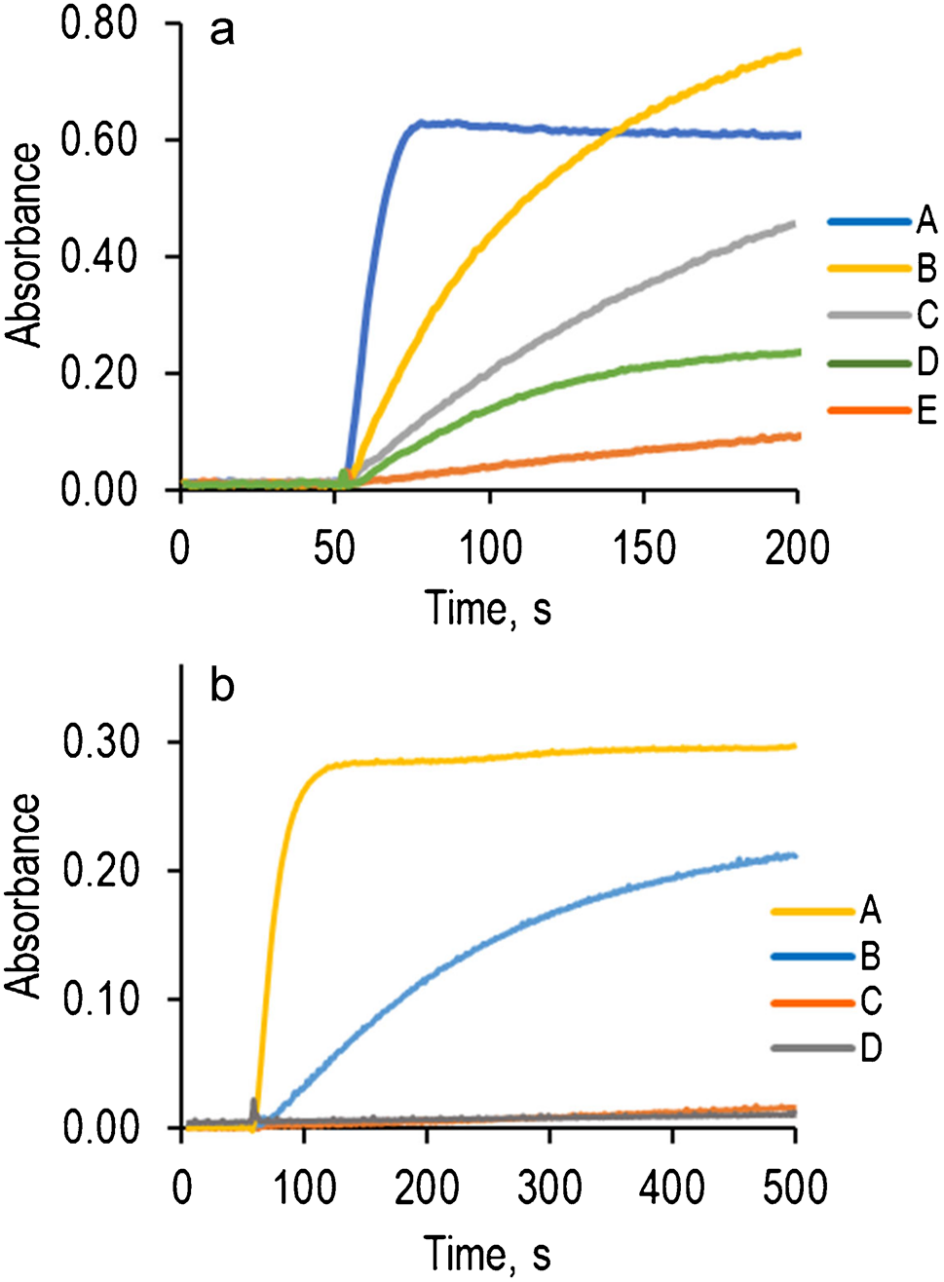
Absorbance variation of cadaverine, putrescine, histamine, and tyramine at different pH. **a** DAO enzymatic reaction. Experimental conditions: [AR] = 5·10^−5^ M, [HRP] = 0.5 U mL^−1^, [DAO] = 1.0 U mL^−1^, [Histamine] = [Tyramine] = [Cadaverine] = [Putrescine] = 5·10^−6^ M, *λ* = 570 nm; (A) cadaverine and putrescine, pH = 7, 8, 9; (B) tyramine, pH = 9; (C) tyramine, pH = 8; (D) histamine, pH = 8, 9; (E) histamine, pH = 7. **b** TAO enzymatic reaction. Experimental conditions: [AR] = 7·10^−5^ M, [HRP] = 0.1 U mL^−1^, [TAO] = 0.5 U mL^−1^, [Histamine] = [Tyramine] = [Cadaverine] = [Putrescine] = 5·10^−6^ M, *λ* = 570 nm; (A) tyramine, pH = 6, 7, 8, 9; (B) histamine, pH = 8, 9; (C) cadaverine and putrescine, pH = 6, 7, 8, 9; (D) histamine, pH = 7

Although DAO shows better analytical figures of merit than TAO, it produces worse selectivity. Figure 4a shows the absorbance vs time representations obtained for the same concentrations of cadaverine, putrescine, tyramine and histamine at the most representative pH using DAO. This confirms its higher sensitivity to putrescine and cadaverine at any pH, but both greatly interfere in the histamine determination; the interference caused by tyramine is also important at different pH values. Figure 4b shows the results obtained with TAO. Putrescine and cadaverine do not interfere at all at any pH and tyramine, as expected, gives the higher signals. Histamine gave good sensitivity at pH = 8, 9 but a very low signal at pH = 7.

It is important to highlight here that depending on the sample to be analyzed, the relative concentrations found of the different BAs change. In general, the most abundant BAs are putrescine and cadaverine, while tyramine appears at low concentrations in different samples (but it is important in cheese samples); finally, the histamine concentration is important in many samples and is very high in scombrids, especially if they are spoiled. So, the method based on TAO/AR/HRP seems to be more suitable than that based on DAO for determining histamine in many types of samples.

#### Simultaneous determination of histamine and tyramine

As illustrated in Fig. 4b, for samples containing relevant concentrations of histamine and tyramine, the method based on TAO provides several possibilities for the simultaneous determination of both analytes, based on the different kinetic behaviors of both BAs at different pH. Here, a two-step method is proposed. The first step consists of measuring the mixture at pH = 7; histamine hardly reacts with TAO, so tyramine can be determined without interference. After that, the histamine concentration can be obtained by measuring the sample at pH = 8. To do this, after removing the contribution of tyramine, the standard addition method was applied (see ESM, Fig. S9 for a detailed explanation). To test this methodology, a synthetic sample containing 8.0·10^−6^ M histamine and 4.1·10^−6^ M tyramine was prepared, and the results obtained (*n* = 3) were 8.1 (± 0.2)·10^−6^ M histamine and 3.9 (± 0.3)·10^−6^ M tyramine, respectively.

### Smartphone-based disposable biosensors

#### Biosensor optimization. Analytical figures of merit

The results obtained with TAO were promising enough to address the development of biosensors sensitive to histamine. These biosensors are intended to be used on-site; the AR_ox_ concentration will be measured using RGB coordinates and a smartphone. Considering the AR_ox_ molecular absorption spectrum and the spectra of the R, G and B filters (see ESM, Fig. S10), the G coordinate was considered the most sensitive. The analytical parameter used throughout the work was ΔG = G_0_ − G. In a previous paper [31], the relationship between ΔG and the analyte (H_2_O_2_ or histamine, depending on the study) concentration (C) is given by:

2 $$\Delta G= {{K}_{2}{C}^{2}+K}_{1}C+ {K}_{0}$$

*K*_0_, *K*_1_, and *K*_2_ are constants which depend on the scattering coefficient of the solid support, the average molar absorptivity of AR_ox_ in the wavelength range of the green coordinate, and the reagents participating in the enzymatic reaction (Appendix 1 describes these values in detail).

The biosensors were built on the same basis as those developed in a previous paper for the determination of cadaverine and putrescine [31]. Cellulose microcrystalline of 20-μm particle size was used. First, the indicating reaction (H_2_O_2_/AR/HRP) was studied. The effect of the cellulose concentration in the mother solution (3 or 5%, w/V) and the HRP and AR concentrations were studied for this particular indicating reaction. From the results obtained, the best experimental conditions to prepare the H_2_O_2_ sensors were achieved (5% cellulose, 0.2 U HRP (Table S2) and from 1.5·10^−9^ mol AR per biosensor (Fig. S11)), and the analytical figures of merit were evaluated (Table 2). As can be seen (Table S2), the G coordinate is the most sensitive. The second-degree polynomial response ranges from 1·10^−5^ M to 5·10^−4^ M. Fig. S12 shows the calibration line and the corresponding colors of the biosensors (before and after the reaction).

**Table 2:** Analytical figures of merit for histamine and tyramine calibration on cellulose supports and smartphone measurement. Experimental conditions: [HRP] = 0.2 U, [AR] = 1.5·10^−8^ mol, [TAO] = 0.25 U

	Calibration line ΔG = K_2_C^2^ + K_1_C + K_0_	Range, M	Calibration line ΔG = K_1_C + K_0_	Range, M	LoD, M	RSD % (*n*)
H_2_O_2_	K_2_ = − 7.0·10^8^; K_1_ = 5.9·10^5^; K_0_ = 4.0	1·10^−5^–5·10^−4^	K_1_ = 5.5·10^5^; K_0_ = 3.8	1·10^−5^–1·10^−4^	3.4·10^−6^	4% (3)
Histamine (pH = 9)	K_2_ = − 3.0·10^8^; K_1_ = 3.7·10^5^; K_0_ = − 0.1	2·10^−5^–5·10^−4^				5% (3)
Histamine, pH = 8	K_2_ = − 2.0·10^8^; K_1_ = 2.7·10^5^; K_0_ = − 0.1	2·10^−5^–5·10^−4^	K_1_ = 2.7·10^5^; K_0_ = − 0.9	2·10^−5^–1·10^−4^	7.5·10^−6^	5% (3)
Tyramine, pH = 8, 30 s			K_1_ = 3.7·10^5^; K_0_ = 0.8	2·10^−5^–1·10^−4^	5.5·10^−6^	5% (3)
Tyramine, pH = 8, 4 min	K_2_ = − 7.0·10^8^; K_1_ = 5.3·10^5^; K_0_ = 0.8	2·10^−5^–5·10^−4^	K_1_ = 4.6·10^5^; K_0_ = 2.0	2·10^−5^–1·10^−4^	5.5·10^−6^	5% (3)

*LoD* limit of detection, *RSD* relative standard deviation

Based on the results obtained in solution, TAO was chosen as the enzyme to build the biosensors for histamine determination. Previous assays indicated that it is better not to use entrapped TAO because it is not very stable (it is probably partially modified during the curing of the biosensors), so it has to be added once the cellulose supports has been set. The results obtained during TAO optimization (Fig. S13) show that the enzyme behaves according to expectations, i.e., the higher the amount of TAO, the lower the response time. The final color is stable so the lateral reaction of AR_ox_ with the outer TAO amino acids (see (1)) has been avoided (just as happened in solution). 0.25 units per biosensor was chosen as the appropriate amount since the signal is at a maximum, the response time is suitable for a fast method (180 s until a stable signal is achieved), and the amount of the reagent consumed is low. Finally, the pH was studied (Fig. S14) again to find the optimal signals and to avoid interferences. The results were similar to those obtained in solution: regarding histamine (Fig. S14a), at pH 5 and 6, it does not react; at pH 7, a small signal is observed, although it is low; and the best results were obtained at pH 8 and 9. Cadaverine and putrescine did not react at any of the tested pH (5–9) and tyramine (Fig. S14b) gave optimal responses at pH 7, 8 or 9.

Lifetime is a very important parameter that describes the biosensor behavior. To study it, cellulose supports were made including the dye and, once dried, they were stored in the dark and refrigerated. The supports were measured over three weeks following the described procedure. Namely, 10 μL of the enzyme mixture was added and the coordinates R_0_,G_0_,B_0_ were taken; after that, 10 μL of a histamine solution was added and the RGB coordinates were taken again after 4 min. Under the optimal conditions chosen, the lifetime was at least 3 weeks (Fig. S15). A similar study was carried out entrapping the enzyme along with the dye, but in this case the biosensors were only stable during 3 days.

The analytical figures of merit were obtained in the optimized conditions (Fig. S16). The response range, sensitivity, LoD and RSD for histamine are shown in Table 2. Again, a second-degree polynomial response range was obtained from 2·10^−5^ M to 5·10^−4^ M, with a linear relationship for low concentrations (from 2·10^−5^ to 1·10^−4^ M).

#### Biosensors for histamine and simultaneous histamine/tyramine determination

The results obtained indicated that these biosensors can be used for in situ histamine determinations in samples having low tyramine concentrations (compared to histamine). However, as occurred in solution, the results allow several methods to be designed for simultaneous histamine and tyramine determination. In this case, since the final aim is to provide a fast and simple method, it was considered more appropriate to take advantage of the different reaction kinetic of both analytes at pH = 8. The tyramine reaction is completed in less than 30 s but the histamine reaction is completed after 4 min. The kinetic profiles given in Fig. S13 show that tyramine can be determined almost without histamine interference during the first seconds of the reaction (30 s), tyramine + histamine can be determined from the final signal and the histamine concentration can be obtained by difference. In order to test this methodology, 6 synthetic samples, containing different histamine/tyramine proportions, were analyzed. To do this determination, histamine and tyramine calibrations were performed at pH = 8. For histamine, the signals were taken 4 min after the injection of the analyte and, for tyramine, they were taken at 30 s and 4 min. The calibration equations obtained are shown in Table 2. To simplify the calculations, the linear Eq. (4th column) was used for quantitative purposes.

**Fig. 5:**
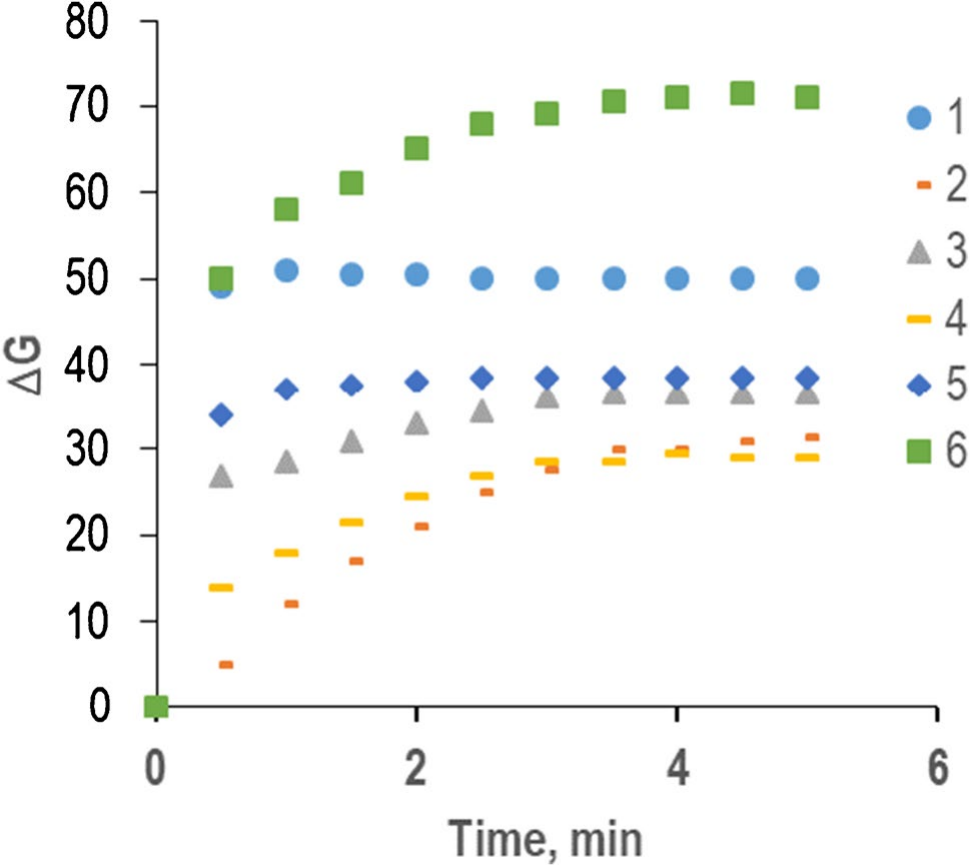
Simultaneous determination of histamine and tyramine. Experimental conditions: cellulose 5% (m/v), [TAO] = 0.23 U, [HRP] = 0.2 U, [Amplex®Red] = 1.5·10^−8^ mol; (1) 1·10^−4^ M tyramine, (2) 1·10^−4^ M histamine, (3) 5·10^−5^ M tyramine + 5·10^−5^ M histamine, (4) 2.5·10^−5^ M tyramine + 7.5·10^−5^ M histamine, (5) 7.5·10^−5^ M tyramine + 2.5·10^−5^ M histamine, (6) 1·10^−4^ M tyramine + 1·10^−4^ M histamine

Table 3 gives the composition of 6 synthetic samples (third column) and the results obtained (fourth column) as well as the relationship between concentrations (tyramine:histamine) in each sample (second column); the experimental ΔG profiles are shown in Fig. 5. As can be seen, when tyramine predominates (samples 1 and 5), the signal rises very fast and it is stable from the beginning (from 30 s), but when histamine predominates, the signal increases progressively over time until it stabilizes (samples 2 and 4). At 4 min, all signals are stabilized. Tyramine was determined by interpolating the signal obtained at 30 s in the corresponding calibration line. For histamine determination, first, the signal obtained with the mixture at 30 s was subtracted from that obtained for the sample at 4 min; the remaining value was interpolated in the corresponding calibration line for histamine.

**Table 3:** Simultaneous determination of histamine and tyramine on cellulose supports and smartphone measurement. Experimental conditions: [HRP] = 0.2 U, [AR] = 1.5·10^−8^ mol, [TAO] = 0.25 U

Sample	Ratio tyramine:histamine	Real concentration	Calculated concentration	Error
1	100:0	1·10^−4^ M tyramine	1.02·10^−4^ M tyramine	2%
		-	-	-
2	0:100	-	-	-
		1.0·10^−4^ M histamine	1.1_1_·10^−4^ M histamine	11%
3	50:50	5.0·10^−5^ M tyramine	5.4_5_·10^−5^ M tyramine	9%
		5.0·10^−5^ M histamine	3.9_5_·10^−5^ M histamine	− 21%
4	25:75	2.5·10^−5^ M tyramine	2.6_2_·10^−5^ M tyramine	5%
		7.5·10^−5^ M histamine	6.1_3_·10^−5^ M histamine	− 18%
5	75:25	7.5·10^−5^ M tyramine	6.9_7_·10^−5^ M tyramine	− 7%
		2.5·10^−5^ M histamine	2.1_4_ ·10^−5^ M histamine	− 14%
6	100:100	1.0·10^−4^ M tyramine	1.0_4_·10^−4^ M tyramine	5%
		1.0·10^−4^ M histamine	7.9_0_·10^−5^ M histamine	− 21%

Using this method, histamine and tyramine were determined with errors ranging from 2 to 21% for both amines (fifth column), which can be considered semiquantitative but fulfilled the objective of having a biosensor for the simple and fast determination of both BAs.

The method was finally applied to the histamine and tyramine determination in a tuna sample (see the “Tuna sample treatment” section). The result obtained (found ± sd) was 972 (± 40) mg kg^−1^ and 240 (± 10) mg kg^−1^ (*n* = 3) respectively. As we indicated in the “Tuna sample treatment” section, the values found by the validated method were 900 (± 40) mg kg^−1^ histamine and 300 (± 22) mg kg^−1^ tyramine, which supposes a relative error of 8% and − 20%, within the order of a semiquantitative method.

Table S3 compares the analytical figures of merit obtained using this biosensor with those obtained using other commercially available tests for histamine. The limit of detection is similar to that obtained with the most sensitive of the commercial tests found but faster, and allows the simultaneous determination of histamine and tyramine, which it has not been reported in the commercial tests developed to date.

## Conclusions

Amplex Red is the appropriate dye for the enzymatic determination of histamine because it prevents the lateral reactions (i.e., oxidation by the aldehyde) observed with other commonly used dyes such as TMB or ABTS.

DAO and TAO are suitable enzymes for determining histamine. DAO is more sensitive; however, when cadaverine and/or putrescine is present in the samples, TAO is more appropriate because it avoids interference.

Although tyramine also reacts when the enzymatic system for histamine is used, it has been shown that it is possible to make a simultaneous semiquantitative determination of both amines.

Biosensors developed by immobilizing AR, HRP, and TAO on cellulose allow the single determination of histamine or simultaneous histamine and tyramine determination in a concentration range from 2·10^−5^ to 5·10^−4^ M.

Regarding the measurement of color, the G coordinate is the most sensitive. Measurements with a smartphone allow the methodology to be fully portable and accessible to any user without specific training. Moreover, the dimensions of the biosensors developed are small and the consumption of reagents is low, which is in line with green chemistry principles.

## Supplementary Information

Below is the link to the electronic supplementary material.

Supplementary file1 (DOCX 1263 KB)
