# Supplementary material for: Portable colorimetric enzymatic disposable biosensor for histamine and simultaneous histamine/tyramine determination using a smartphone
Source: Anal Bioanal Chem. 2023 Feb 15;415(9):1777–86. doi: 10.1007/s00216-023-04583-0 (PMC9992026; doi:10.1007/s00216-023-04583-0)
Supplement: Supplementary file 1 — Supplementary file1 (DOCX 1263 KB) [file 216_2023_4583_MOESM1_ESM.docx]

**SUPPLEMENTARY MATERIAL**

**PORTABLE COLORIMETRIC ENZYMATIC DISPOSABLE BIOSENSOR FOR HISTAMINE AND SIMULTANEOUS HISTAMINE/TYRAMINE DETERMINATION USING A SMARTPHONE.**

I. Sanz-Vicente^a^, I. Rivero^b^, L. Marcuello^b^, M.P. Montano^b^, S. de Marcos^a^ and J. Galbán^a^.

a.- Nanosensors and Bioanalytical Systems (N&SB), Analytical Chemistry Department, Faculty of Sciences, Aragon Institute of Nanoscience, University of Zaragoza, 50009-Zaragoza, Spain.

b.- Analytical Chemistry Department, Faculty of Sciences, University of Zaragoza, 50009-Zaragoza, Spain.

**Appendix 1**

According to our previous model [ref 31] of the main manuscript] the following equation relates the brightness of G color coordinate with the concentration of the absorbing species (*c*, in M) in the solid:

$$G=A\left( G_{0}- G_{1}c+ G_{2}c^{2} \right) (S1)$$

“A” being a constant which includes different instrumental factors mainly depending on the specific camera used (camera design, solid angle measurement, light to voltage transformation and analog-to-digital conversion), and G_0_, G_1_ and G_2_ are given by:

$$G_{0}=\sum_{\lambda} \left[ I_{\lambda}P_{G,\lambda}U_{\lambda} \right] G_{1}=\sum_{\lambda} \left[ I_{\lambda}P_{G,\lambda}V_{\lambda}\varepsilon_{\lambda} \right] G_{2}=\sum_{\lambda} \left[ I_{\lambda}P_{G,\lambda}{W_{\lambda}\varepsilon}_{\lambda}^{2} \right] (S2)$$

I_λ_ and P_G,λ_ being the spectral power of the illumination source and the camera spectral sensitivity (which is the product of the sensitivity of the CCD and the transmittance of the Bayer filter at the corresponding λ), respectively, ε_λ_ being the molar absorptivity of the absorbing species (M^-1^cm^-1^), and U_λ_, V_λ_ and W_λ_ being parameters depending on the average scattering coefficient ($\underline{s}$, cm^-1^) and the thickness (L, cm^-1^) of the solid support.

Although s_λ_ of the materials depends (slightly) on the wavelength, for the visible region of the spectra an average value is normally used ($\underline{s}$) :

$${U_{\lambda}=U=\frac{\underline{s}L}{1+\underline{s}L} \left( S3 \right) V}_{\lambda}=V=4,6\left( \frac{3+2\underline{s}L}{3\underline{s}} \right)\left( \frac{\underline{s}L}{1+\underline{s}L} \right)^{2} (S4)$$

$$W_{\lambda}=W= \left( \frac{\underline{s}L}{1+\underline{s}L} \right)^{3}\left( \frac{30+45s_{\lambda}L+24\left( s_{\lambda}L \right)^{2}+4\left( s_{\lambda}L \right)^{3}}{{2s}_{\lambda}^{2}} \right) (S5)$$

According to Mie theory, in solid media (high particles) the s_λ_ slightly depends on λ for a moderate wavelength interval, so it can be considered constant ($\underline{s}$) for each coordinate. Moreover, despite ε_λ_ depends on the wavelength, as the detector integrates the whole radiation passing across the filter, a weighted average value ($\underline{\varepsilon_{G}}$) is also representative of the absorbing characteristic of the species. In these conditions U, V, W and ε_λ_ can be considerate constant for the wavelength interval that encompasses each color coordinate. Under these considerations equation (S2) is simplified to:

$$G_{0}=U\sum_{\lambda} \left[ I_{\lambda}P_{G,\lambda} \right] G_{1}=V\underline{\varepsilon}_{G}G_{0} G_{2}=W\underline{\varepsilon}_{G}^{2}G_{0} (S6)$$

And the ΔG is given by:

$$\Delta G=G_{0}-G={(G}_{0}-{AG}_{0})+AV\underline{\varepsilon}_{G}G_{0}c-AW\underline{\varepsilon}_{G}^{2}G_{0}c^{2} (S7)$$

Finally, to relate *c* with the analyte concentration (H_2_O_2_ or Histamine) the kinetic of the enzymatic reactions have to be considered. Optimization on the experimental conditions in solution (see main manuscript page 8) allows indicate that:

*c* = [AR_ox_] = α[H_2_O_2_]_0_ (or α[Histamine]_0_)

α=1 for H_2_O_2_ and 0.63 for Histamine. In general,

$$K_{0}=\alpha{(G}_{0}-{AG}_{0}) K_{1}=\alpha AV\underline{\varepsilon}_{G}G_{0} K_{2}= \alpha AW\underline{\varepsilon}_{G}^{2}G_{0} (S8)$$

After replacing (S8) in (S7) the following equation is obtained

$$\Delta G=K_{2}C^{2}+K_{1}C+ K_{0} (S9)$$

C being H_2_O_2_ or Histamine. This equation can be applied to other BA, but the “α” will be different. Note that K_2_ is negative and K_1_ positive.

**Fig. S1 Measurement system using a Smartphone**

**
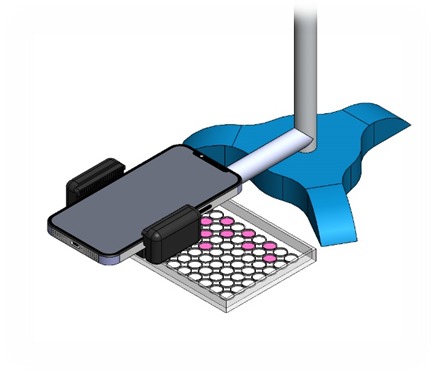
**

The position of the smartphone was fixed at the same height from the samples and the biosensors were moved under it.

**Fig. S2 AR_ox_ molecular absorption spectrum**

This figure shows the molecular absorption spectrum using 2.5·10^-6^ M AR_ox_.


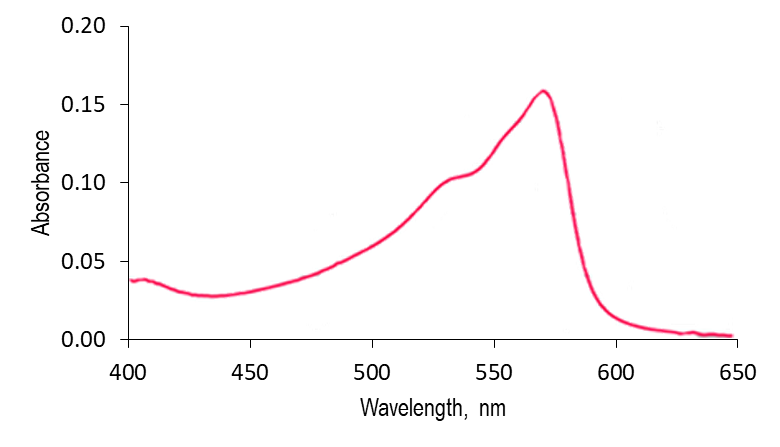


**Fig. S3 Optimization of the AR concentration**

The following figure shows the results obtained. The maximum signal is obtained from 4·10^-6^ M of AR.


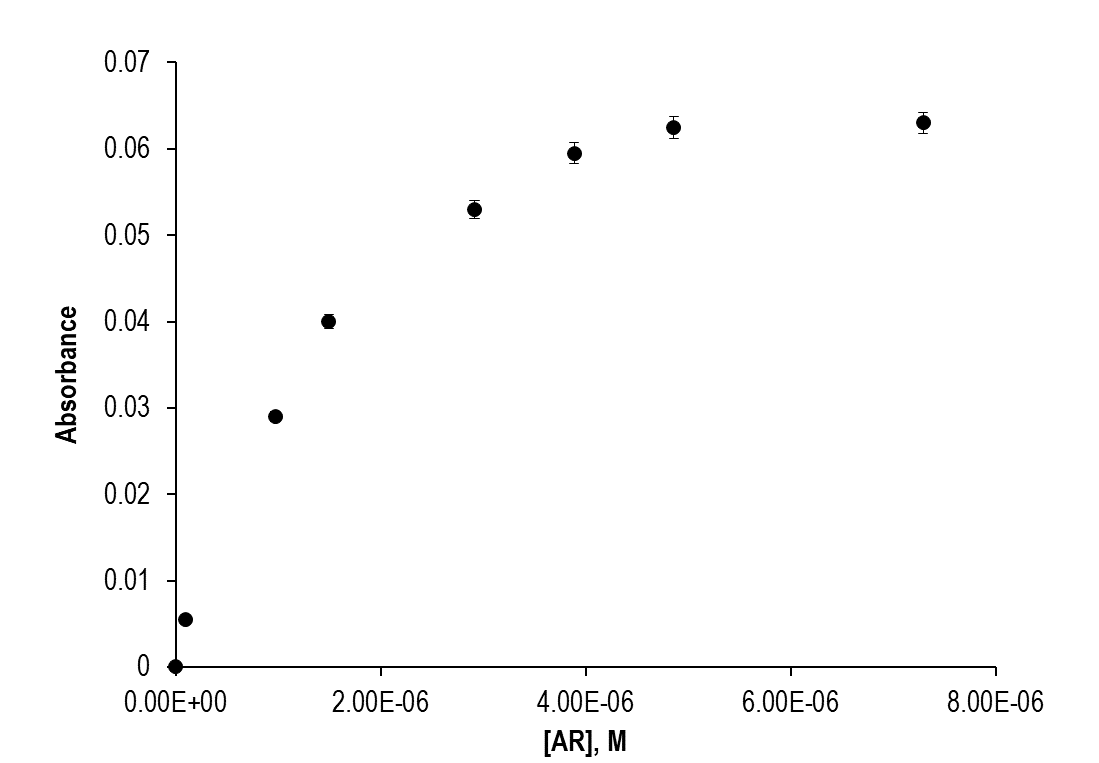


*Experimental conditions: [HRP]=2 U mL^-1^, [H_2_O_2_]= 9.8·10^-7^ M, pH 6,λ=570nm, n=5.*

**Fig. S4 Optimization of the HRP concentration**

As can be seen, the maximum signal is obtained from 0.2 U mL^-1^ of HRP.


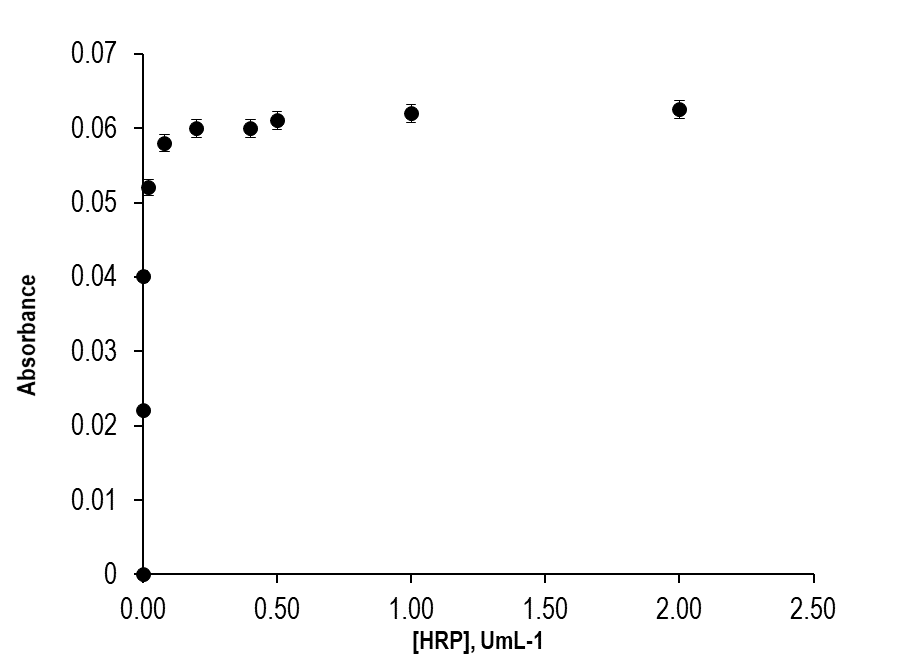


*Experimental conditions: [AR]= 5·10^-6^ M, [H_2_O_2_]= 9.8·10^-7^ M, pH 6, λ=570nm, n=5.*

**Fig. S5 Calibration line of H_2_O_2_.** *Experimental conditions: [AR]= 5·10^5^ M, [HRP]=0.50 U mL^-1^, pH 6, λ=570nm, n=5.*

***
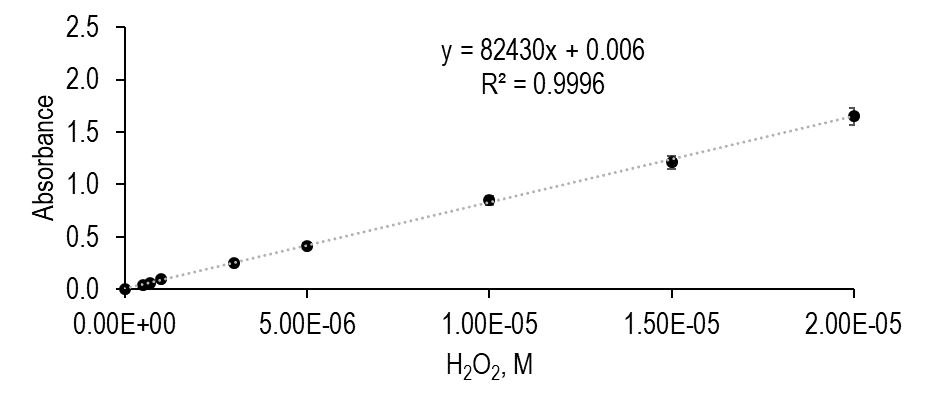
***


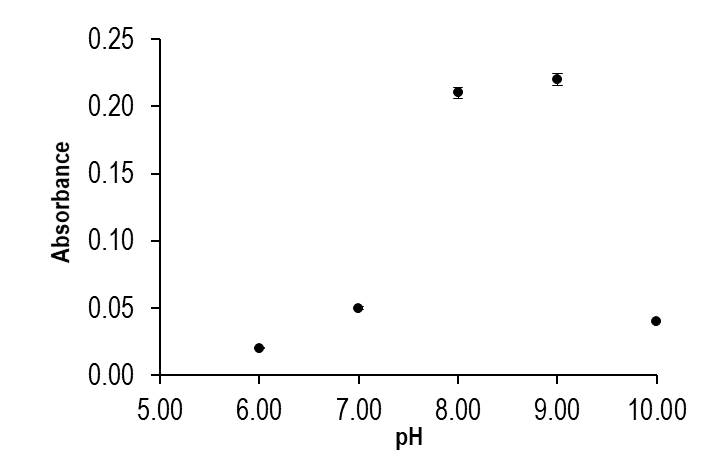

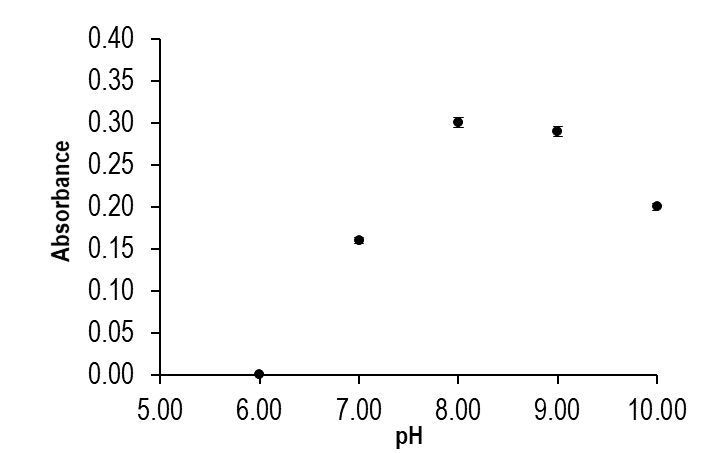
**Fig. S6 pH optimization study. a) [DAO] =1.0 U mL^−1^,** [AR] = 5·10^−5^ M, [HRP] = 0.5 U mL^−1^, [Histamine] = 5·10^−6^ M, λ= 570 nm*, n=5* **b)** **[TAO] =0.5 U mL^−1^,** [AR] = 7·10^−5^ M, [HRP] = 0.1 U mL^−1^, [Histamine] = 5·10^−6^ M, λ= 570 nm, *n=5*

**a**

**b**

**Table S1 Effect of the HRP concentration**.

The HRP concentration affects the initial rate of the reaction. Since the HRP is not very important, it can be deduced that amine oxidase is the enzyme that controls the reaction.

| HRP, U mL^−1^ | 0.01 | 0.03 | 0.10 | 0.25 | 0.50 | 1.0 | 2.0 |
| --- | --- | --- | --- | --- | --- | --- | --- |
| Initial rate, Abs s^-1^ | 1.68±0.03 | 1.88±0.03 | 2.06±0.04 | 2.02±0.04 | 1.98±0.04 | 1.96±0.04 | 1.95±0.04 |

Experimental conditions: [AR] = 7*·*10^−5^ M, [DAO] = 2 U mL^−1^, [Histamine] = 5*·*10^−6^ M, λ= 570 nm**,** pH=8, n=3

**Fig. S7 Effect of the AR concentration**.

The optimum **AR** to Histamine concentrations ratio was studied between from 2:1 and 18:1. As can be seen, AR does not affect the signal obtained very much. Considering the results given in Fig. S3, 7·10^-5^ M was final chosen.

| AR, M | 9·10^-6^ | 2·10^-5^ | 5·10^-5^ | 7·10^-5^ | 9·10^-5^ |
| --- | --- | --- | --- | --- | --- |
| Absorbance±sd | 0.250±0.005 | 0.252±0.005 | 0.263±0.005 | 0.267±0.005 | 0.268±0.005 |
| [AR]:[Histamine] | 2:1 | 5:1 | 10:1 | 14:1 | 18:1 |

Experimental conditions: [HRP] = 0.1U mL^-1^, [DAO] = 2 U mL^−1^, [Histamine] = 5·10^−6^ M, λ= 570 nm**,** pH=8, n=3

**Fig. S8 Histamine calibration lines using DAO (a, 2 U mL^−1^) and TAO (b, 1 U mL^−1^).** Experimental conditions: [HRP] = 0.1 U mL^-1^, [AR]= 7·10^−5^ M, λ= 570 nm**,** pH=8, n=3


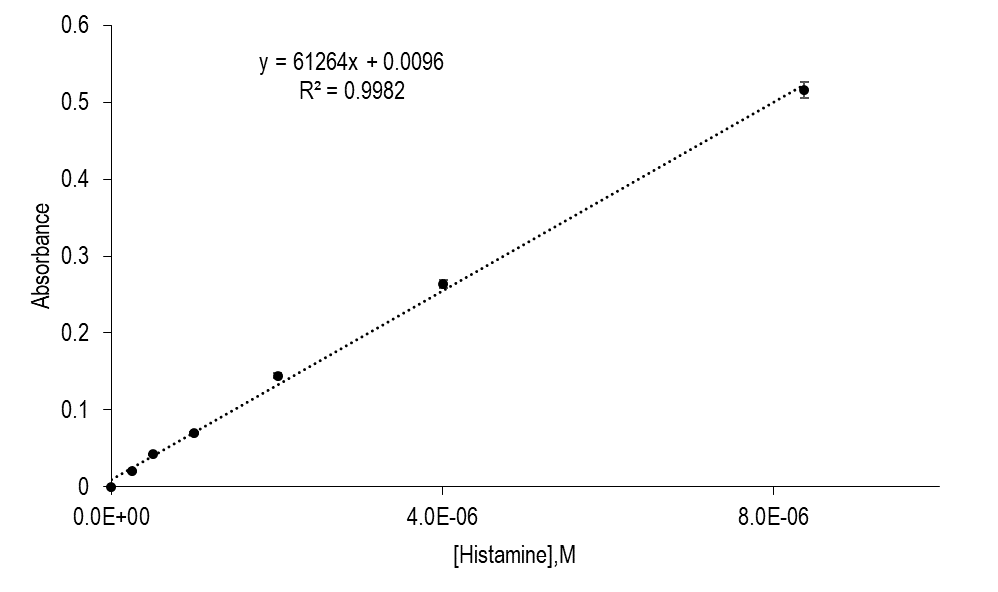
**a)**


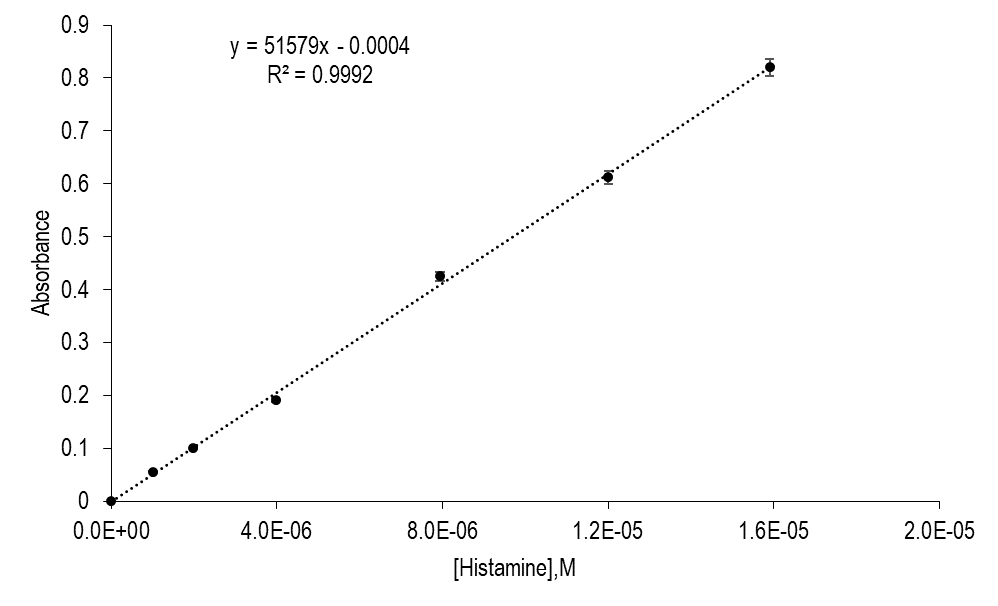
**b)**

**Fig. S9 Simultaneous determination of Histamine and Tyramine.**

The measurements were carried out using the optimized parameters.

First, the concentration of tyramine at pH 7 is obtained. To do this, a tyramine calibration study was performed with increasing concentrations up to 8·10^-6^ M. Later, the sample was measured (in triplicate) and the average signal was interpolated in the tyramine calibration line, obtaining 3.9 (±0.3)·10^-6^ M. With that concentration, a tyramine standard was prepared and measured at pH 8 to determine the absorbance at that pH.

Second, histamine concentration was obtained using the standard addition method (added concentrations: 0.4·10^-6^, 8·10^-6^ and 1.2·10^-5^ M); the absorbance of the 3.9·10^-6^ M tyramine standard was previously subtracted from the signal obtained after the measurement of each standard solution.


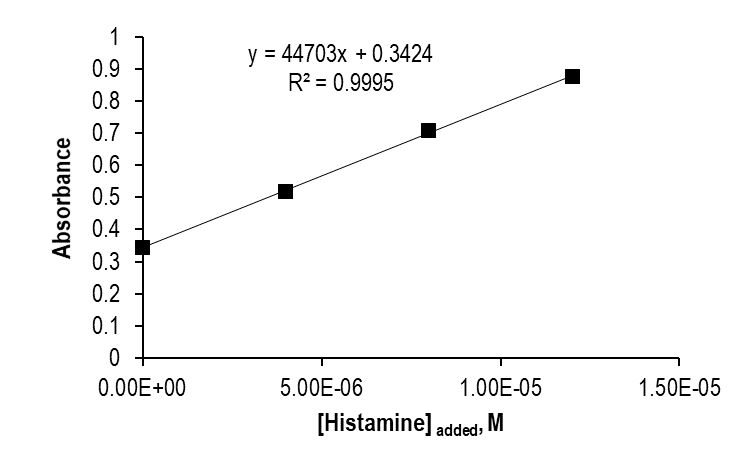


Experimental conditions: [HRP] = 0.1U mL^-1^, [AR]= 7·10^−5^ M, [TAO] = 1 U mL^−1^, λ= 570 nm**,** pH=8

The Histamine concentration was calculated obtaining 8.1(±0.2)·10^-6^ M.

**Fig. S10 Spectra of R, G, B filters and AR_ox_.**

The figure (taken from <https://photo.stackexchange.com/questions/87172/can-we-compare-the-color-reproduction-accuracy-of-2-camera-sensors-only-by-looki>) shows the red, green and blue sensors quantum efficiency (QE) of a Sony IMX249 camera. The orange line corresponds to the AR_ox_ absorption spectrum. As can be seen, the wavelength range of the AR_ox_ absorption band (570 nm) fits very well with the G sensor wavelength range.**
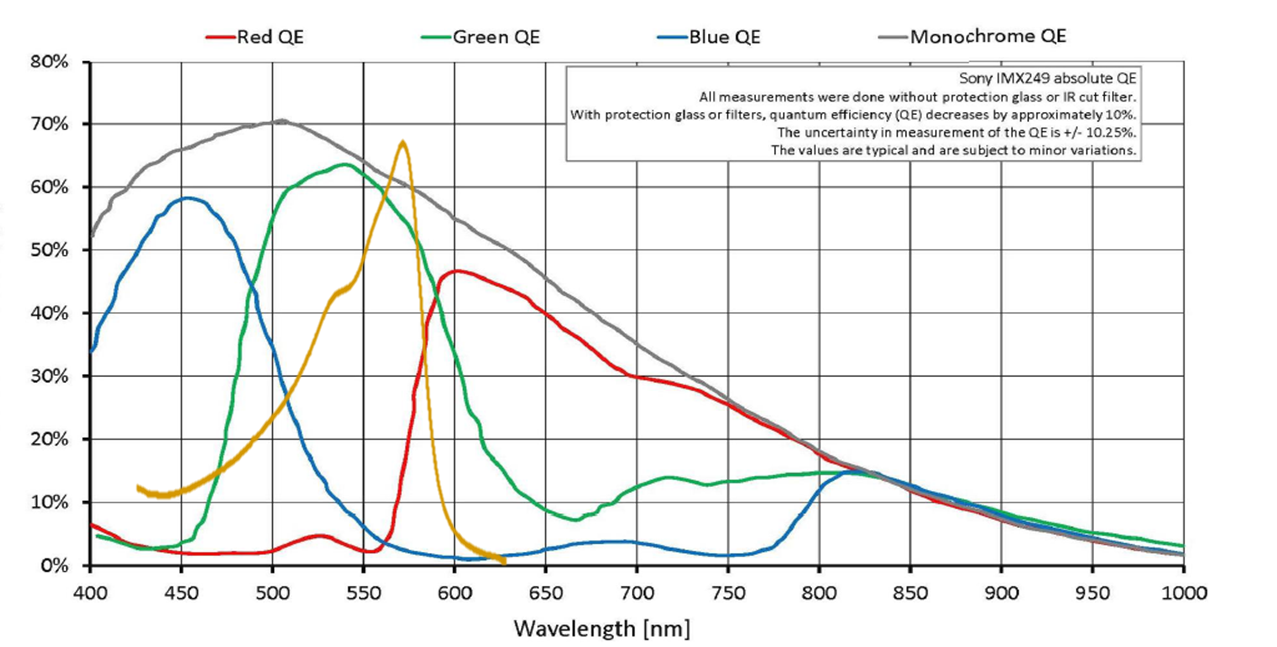
**

**Table S2 Optimization of the biosensors to the indicating reaction H_2_O_2_/HRP/AR**

The effect of the concentration of cellulose and the quantity of HRP per biosensor was studied.

|  | Cellulose 3 % (m/v) | | | Cellulose 5 % (m/v) | | |
| --- | --- | --- | --- | --- | --- | --- |
|  | Δ R ± σ | Δ G ±σ | Δ B ±σ | Δ R ± σ | Δ G± σ | Δ B ± σ |
| Blank | 12 | 15 | 11 | 7 | 19 | 10 |
| HRP 0.1 U | 10 ± 1.4 | 35 ± 0.7 | 11 ± 0.7 | 2.0 ± 2.8 | 47 ±6.4 | 4.5 ± 6.4 |
| HRP 0.2 U | 3.0 ± 0.7 | 48 ± 0.7 | 13 ± 2.9 | **2.0 ± 2.1** | **60 ± 3.5** | **13 ± 2.1** |
| HRP 0.4 U | 0.0 ± 1.4 | 44 ± 1.4 | 8.0 ± 2.8 | 1.0 ± 1.4 | 56 ± 7.1 | 12 ± 5.7 |
| HRP 0.8 U | 4.0 ± 4.9 | 50 ± 4.9 | 16 ± 2.1 | 4.0 ± 2.1 | 54 ± 2.1 | 6.0 ± 2.1 |

Experimental conditions: [AR]= 1.5·10^−8^ mol/biosensor, pH=8, [H_2_O_2_] = 5·10^−5^ M, injection volume= 10 μL, n=3

Cellulose 5 % was chosen. The HRP concentration affects both the signal obtained and the rate of the reaction; 0.2 U per biosensor was considered optimum. The highest sensitivity was obtained using the G component (ΔG).

**Fig. S11 Optimization of the biosensors to the indicating reaction H_2_O_2_/HRP/AR**

The effect of the quantity of **AR was studied**


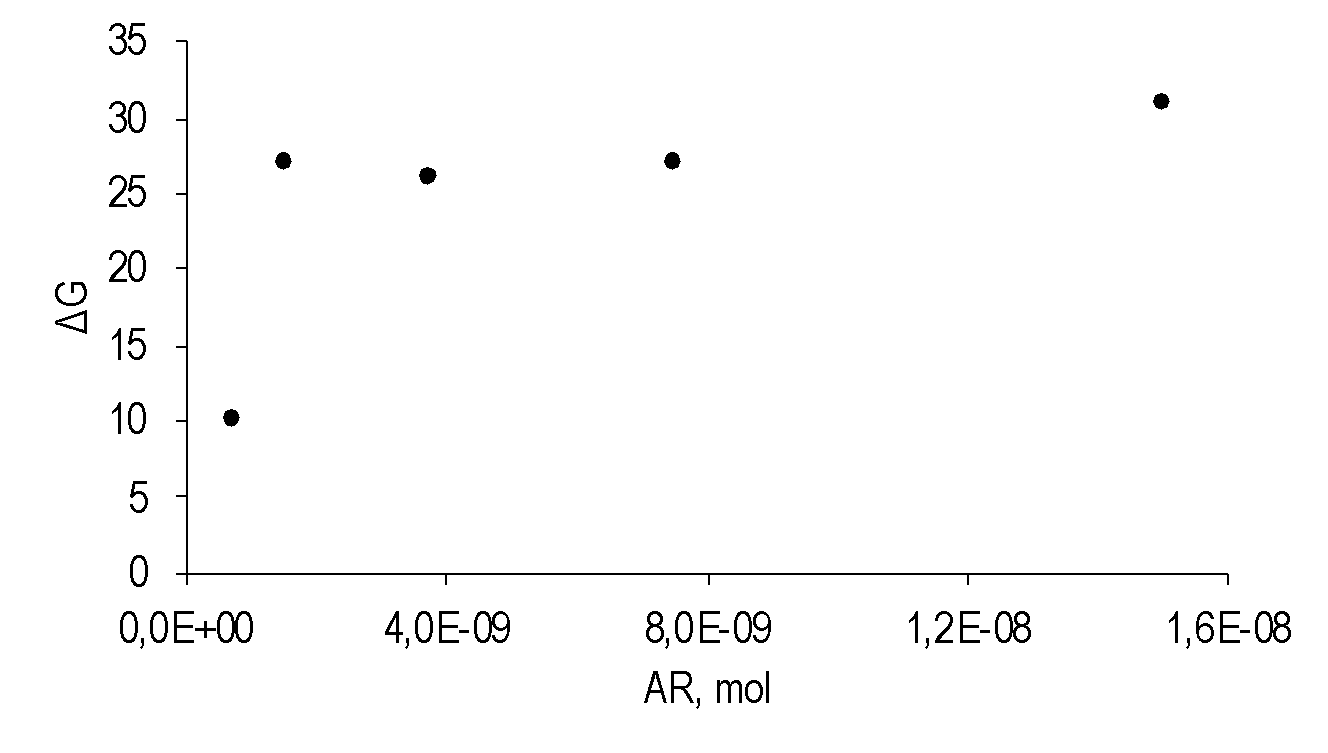


Experimental conditions: [HRP]=0.2 U/biosensor, pH=8, [H_2_O_2_] = 5·10^−5^ M, injection volume= 10 μL

As can be seen, the quantity of AR is optimum from 1.5·10^-9^ mol/biosensor.

**Fig. S12 Calibration of H_2_O_2_ using the biosensors.** Experimental conditions: pH=8, [HRP]=0.2 U/biosensor, [Amplex®Red]= 1.5·10^-8^ mol/biosensor, n=3.

The image on the left shows the supports before adding the analyte. The image on the right shows the supports after the reaction. B: blank, 1: 1·10^-5^ M, 2: 3·10^-5^ M, 3: 5·10^-5^ M, 4: 7·10^-5^ M 5: 1·10^-4^ M, 6: 2·10^-4^ M, 7: 5·10^-4^M


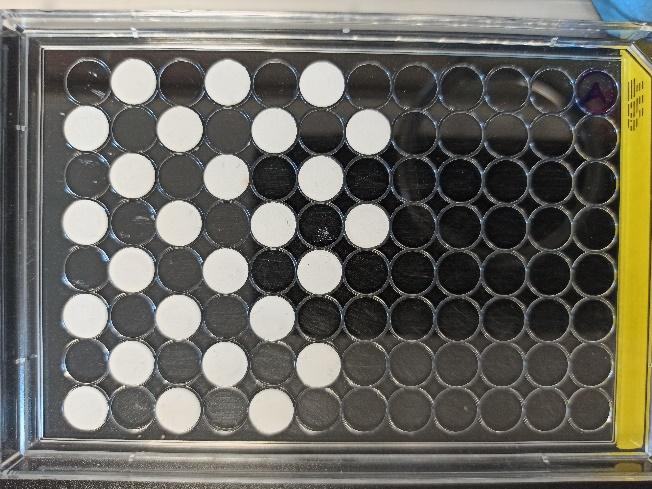

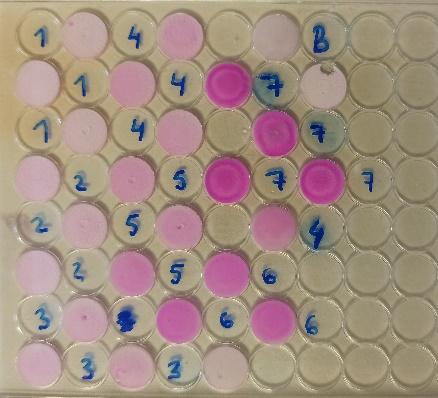


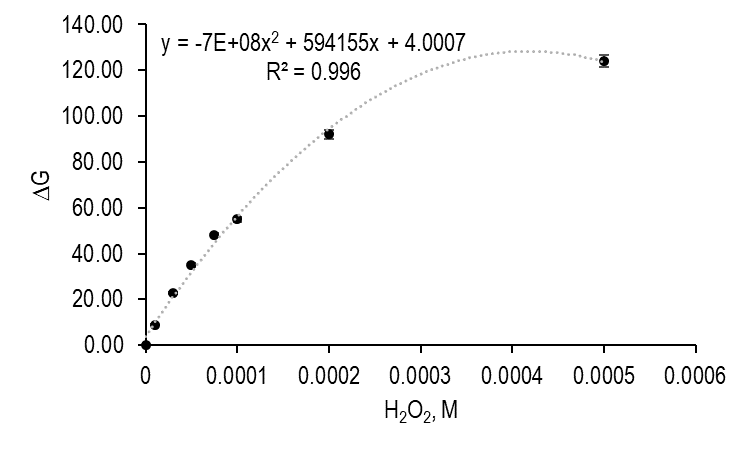


**Fig. S13 Optimization of TAO in the biosensors**. Experimental conditions: pH 8, cellulose 5 % (m/v), [HRP]=0.2 U/biosensor, [AR]= 1.5·10^-8^ mol/biosensor, [Histamine]= 5·10^-5^ M) (blue circles: 0.46 U/biosensor; orange circles: 0.25 U/biosensor; grey circles: 0.12 U/biosensor)


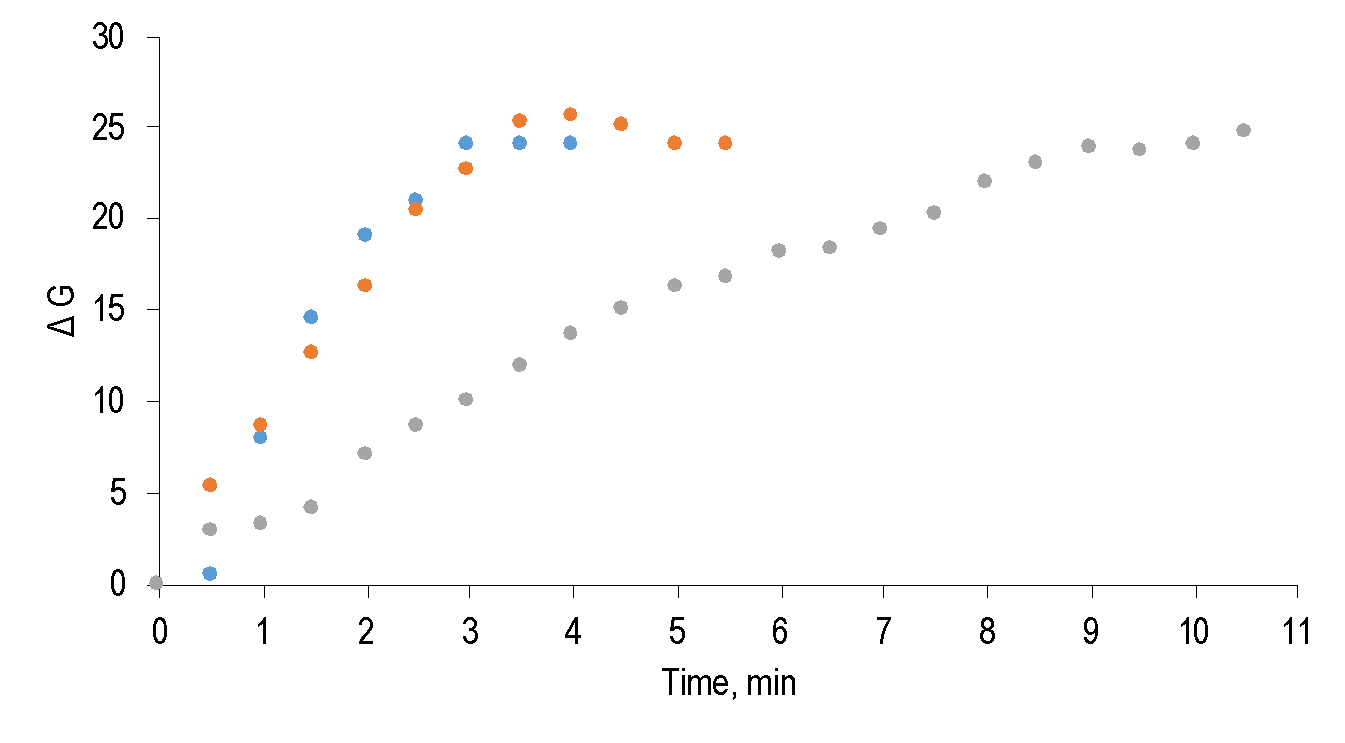


**Fig. S14 Influence of pH inthe signal of a) Histamine b) Tyramine.** Experimental conditions: cellulose 5 % (m/v), TAO=0.12 U/biosensor, [HRP]=0.2 U/biosensor, [AR]= 1.5·10^-8^ mol/biosensor, [Histamine]= 5·10^-5^ M, [Tyramine]= 1·10^-5^ M

a)


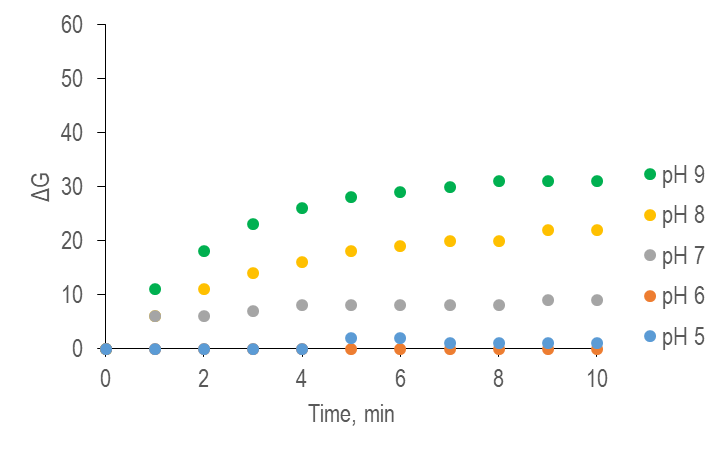


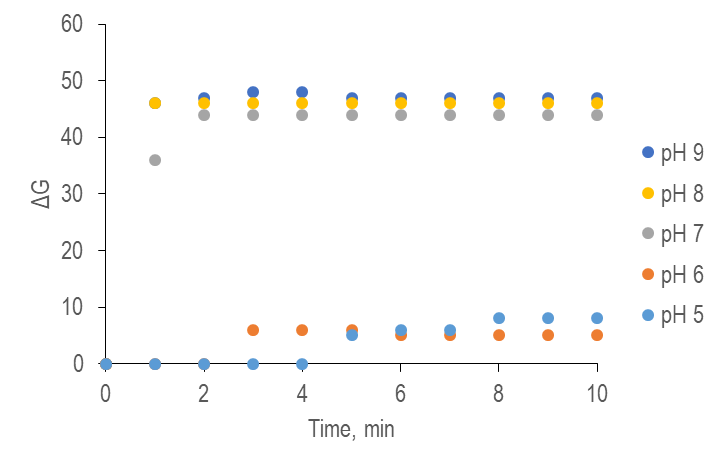
b)

**Fig. S15 Study of the life time of the biosensors.**

Cellulose supports were made including the dye and, once dried, they were stored in the dark and refrigerated. The supports were measured over three weeks following the described procedure (10 microliters of the enzyme mixture was added and the coordinates R_0_,G_0_,B_0_ were taken; after that, 10 microliters of a histamine solution were added and the RGB coordinates were taken again after 4 minutes.) Under the optimal conditions chosen, the lifetime was at least 3 weeks (method 1). A similar study was carried out entrapping the enzyme along with the dye, but in this case the biosensors were only stable during three days (method 2).

Experimental conditions: pH=8, [HRP]=0.2 U/biosensor, [TAO]=0.25 U/biosensor, [Amplex®Red]= 1.5·10^-8^ mol/biosensor, [Histamine]= 2.1·10^-4^M, n=3,

**
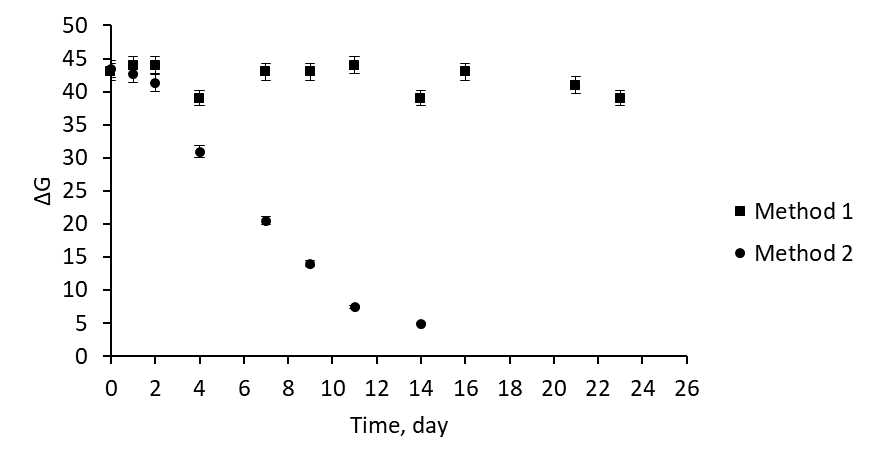
**

**Fig. S16 Calibration of Him using the biosensor.** Experimental conditions: pH=9, [HRP]=0.2 U/biosensor, [TAO]=0.25 U/biosensor, [Amplex®Red]= 1.5·10^-8^ mol/biosensor, n=3, B: blank, 1: 3·10^-5^ M, 2: 5·10^-5^ M, 3: 7.5·10^-5^ M 4: 1·10^-4^ M, 5: 2·10^-4^ M, 6: 5·10^-4^M


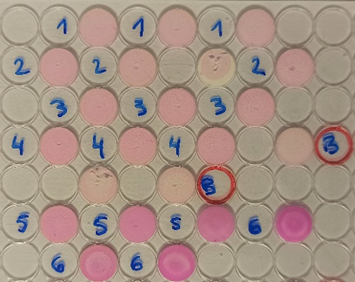


**Table S3 Comparison between the presented method to commercial test.**

| Name | Method | Range, Histamine | LoD, Histamine | Time | Simultaneous determination Histamine+Tyramine | Ref |
| --- | --- | --- | --- | --- | --- | --- |
| Biosensor | Enzymatic | 2.2-55.5 mg/L | 0.83 mg/L | 4 min | yes | This work |
| HISTASURE | ELISA | 3-300 ppm | 0.44 ppm | 5 min acyilation  +ELISA 20 min | no | [1] |
| VERATOX | ELISA | 2.5-40 mg/L | 2 mg/L | 20 min | no | [2] |
| HISTASTRIP | Enzymatic | 125-500 ppm | 50 ppm | 4 min | no | [3] |

LoD: limit of detection

1. <http://www.meridianhealthcare.it/wp-content/uploads/2014/08/Histamine.pdf>, accessed 14 December 2022

2. <https://www.neogen.com/globalassets/pim/assets/original/10021/16142j-veratox-for-histamine-tuna-pack_9506_kitinsert.pdf>, accessed 14 December 2022

3. <https://www.perkinelmer.com/es/product/histastrip-food-1100-01>, accessed 14 December 2022
